# Comparative gut transcriptome analysis of *Diatraea saccharalis* in response to the dietary source

**DOI:** 10.1371/journal.pone.0235575

**Published:** 2020-08-03

**Authors:** Daniel D. Noriega, Fabricio B. M. Arraes, José Dijair Antonino, Leonardo L. P. Macedo, Fernando C. A. Fonseca, Roberto C. Togawa, Priscila Grynberg, Maria C. M. Silva, Aldomario S. Negrisoli, Carolina V. Morgante, Maria F. Grossi-de-Sa

**Affiliations:** 1 Embrapa Genetic Resources and Biotechnology, Brasília-DF, Brazil; 2 Department of Cellular Biology, University of Brasília, Brasília-DF, Brazil; 3 Catholic University of Brasília, Brasília-DF, Brazil; 4 Biotechnology Center, UFRGS, Porto Alegre-RS, Brazil; 5 Departamento de Agronomia/Entomologia, UFRPE, Recife-PE, Brazil; 6 Embrapa Tabuleiros Costeiros, Aracaju, Sergipe-SE, Brazil; 7 Embrapa Semi Arid, Petrolina-PE, Brazil; 8 National Institute of Science and Technology–INCT PlantStress Biotech–EMBRAPA, Brasilia-DF, Brazil; Universidade Federal do Rio de Janeiro, BRAZIL

## Abstract

The sugarcane borer (*Diatraea saccharalis*, Fabricius, *1794*) is a devastating pest that causes millions of dollars of losses each year to sugarcane producers by reducing sugar and ethanol yields. The control of this pest is difficult due to its endophytic behavior and rapid development. Pest management through biotechnological approaches has emerged in recent years as an alternative to currently applied methods. Genetic information about the target pests is often required to perform biotechnology-based management. The genomic and transcriptomic data for *D*. *saccharalis* are very limited. Herein, we report a tissue-specific transcriptome of *D*. *saccharalis* larvae and a differential expression analysis highlighting the physiological characteristics of this pest in response to two different diets: sugarcane and an artificial diet. Sequencing was performed on the Illumina HiSeq 2000 platform, and a *de novo* assembly was generated. A total of 27,626 protein-coding unigenes were identified, among which 1,934 sequences were differentially expressed between treatments. Processes such as defence, digestion, detoxification, signaling, and transport were highly represented among the differentially expressed genes (DEGs). Furthermore, seven aminopeptidase genes were identified as candidates to encode receptors of Cry proteins, which are toxins of *Bacillus thuringiensis* used to control lepidopteran pests. Since plant-insect interactions have produced a considerable number of adaptive responses in hosts and herbivorous insects, the success of phytophagous insects relies on their ability to overcome challenges such as the response to plant defences and the intake of nutrients. In this study, we identified metabolic pathways and specific genes involved in these processes. Thus, our data strongly contribute to the knowledge advancement of insect transcripts, which can be a source of target genes for pest management.

## Introduction

The relationship between herbivorous insects and their hosts has given rise to one of the most complex co-evolutionary processes in natural history. Plant-insect interactions have resulted in a broad spectrum of adaptive responses in both hosts and insects [1]. For a phytophagous insect, a suitable host provides its nutritional requirements and, at the same time, allows the insect to avoid risks from the environment. However, in most cases, the host plant develops mechanical and chemical barriers that pose a risk to insect survival.

Thus, the success of phytophagous insects relies on their ability to overcome these challenges [2]. Plant chemical defences are diverse, including phytohormones, secondary metabolites, proteinaceous defences, and volatile compounds [3, 4]. Based on these characteristics, ecologists classified herbivorous insects following the traditional specialist-generalist dichotomy. Generalists are polyphagous and exhibit a broad pool of mechanisms to efficiently degrade and excrete toxic compounds produced by a wide range of hosts. On the other hand, specialists are restricted to a narrow range of hosts, though some of them possess sequestration mechanisms that confer them with the ability to use the defence traits of a given host in their favour [5]. For specialists, the production of digestive and transporter molecules is a fundamental process that allows them to exploit nutrients from plant material optimally [6].

In holometabolous herbivorous insects, particularly those whose feeding strategy is chewing, the larval stages present more active feeding behavior. Furthermore, the major organ in larval phases is the gut (especially the midgut portion), which plays an essential role in digestion, nutrient absorption, and detoxification [7, 8]. In the last decade, a significant number of studies have revealed the expression profiles of many genes involved in such processes by using different omics tools. In almost all cases, the larval gut has been shown to be the tissue in which the regulation of these genes is critical for host selection, adaptation to plant defences, and insecticide resistance [2, 9]. High-throughput technologies for obtaining genomic and transcriptomic data are becoming more accessible, allowing the identification of potential pest management targets. High-quality transcriptomes are powerful tools for designing pest management strategies and are required for biotechnological approaches [10–14]. Candidate receptors for *Bacillus thuringiensis* (*Bt*) toxins and target genes for gene silencing by RNA interference are some of the findings from these studies [15, 16].

The sugarcane borer, *Diatraea saccharalis* (Fabricius, 1794) (Lepidoptera: Crambidae), is a specialist herbivorous and a devastating pest that exhibits chewing behavior [17]. It causes economic impacts in sugarcane crops across the American continent [18, 19], especially in Brazil, the world’s largest sugarcane producer, where infestations interfere with the quality of the raw material and reduce the yield and quality of the sugar and ethanol [20]. The available genomic data of *D*. *saccharalis* are very limited, and no public genome has been published to date. The first transcriptome of *D*. *saccharalis* was recently released, revealing the regulation of some key metabolic and developmental pathways by the larval endoparasitoid *Cotesia flavipes* [21]. Here, we investigated the intestinal transcriptome profile of *D*. *saccharalis* larvae, providing a tissue-specific, high-quality sequence database for gut metabolism. Besides, the expression profile of candidate target genes was assessed in response to different dietary conditions to provide a better understanding of how metabolism is affected by the food source. Here, we outline profiles of transcriptomic variations to highlight potential target genes for the development of control strategies to be used against this important insect pest.

## Materials and methods

### Isolation of larval gut

Eggs of *D*. *saccharalis* were provided by Fitoagro Controle Biológico Ltda. from a lab colony in Maceio-AL (Brazil). Larvae were maintained on an artificial diet [22] up to the third instar and subsequently starved for 24 h. Then, larvae with the same body size and weight were subjected to three different treatments: starvation for two days, feeding on sugarcane thatch and feeding on artificial diet for five days. Temperature of 26 ± 1 C°, humidity of 65 ± 5 and a 12:12 light:dark cycle were maintained continuously. To obtain isolated guts, the larvae were washed with distilled water to remove any traces of vegetal material. Then, each larva was placed into a petri dish (100 mm × 15 mm) containing 10 mL of NaCl 0.9% solution. The dissection was performed using a scalpel, by cutting crosswise into the last segment of the body’s posterior region. After, the entire gut was kindly removed with entomological forceps and by pressing the anterior region of the larva in order to favour contractions that gradually released the gut towards the posterior end. The attached Malpighian tubes were removed, and any remaining food inside the gut was drained. Lastly, isolated guts were placed briefly into filter paper (12.5 cm) to drain the excess of saline solution.

### RNA sample preparation and sequencing

Isolated guts from each group were frozen in liquid nitrogen, and total RNA was extracted using the TRIzol reagent kit (Invitrogen, Carlsbad, CA, USA). The RNA's quality was assessed with a 2100 Bioanalyzer Instrument (Agilent, Santa Clara-CA, USA). The quantification of RNA was performed by using the Qubit RNA BR Assay Kit (Invitrogen, Carlsbad, CA, USA). Finally, two independent sample replicates per treatment were sequenced by the Illumina HiSeq 2000 platform (Illinois, USA), using RNA extracted from a pool of 20 larval guts. The cDNA library construction was performed to produce paired-end reads (PE reads) with 100 bp each. All procedures followed the manufacturer’s instructions.

### *De novo* assembly and functional annotation

Illumina libraries were constructed using “TruSeq Stranded RNA-Seq Sample Prep Kit”. The libraries were pooled in equimolar concentration and quantitated by qPCR on one lane using a “TruSeq Rapid SBS Sequencing Kit v2”. Fastq files were generated and demultiplexed with the bcl2fastq v1.8.4 (Illumina). Adaptors were clipped, and low-quality raw sequences (Phred < 28) were trimmed using Trimmomatic v0.33 [23]. For assembly, the reads were digitally normalized using KHMER v2.0 [24] with maximum coverage of 50 for each read. Next, the processed reads were *de novo* assembled into contigs using Trinity v2.0.6 [25], using specific parameters (SS_lib_type RF and min_contig_length 300), thus generating a set of components (unigenes) containing a variable number of transcripts (isoforms). All contigs were subjected to Blast searches against the National Center for Biotechnology Information (NCBI) non-redundant protein (NR) database using the BlastX algorithm with an e-value cut off of < 1E-5 (https://blast.ncbi.nlm.nih.gov/Blast.cgi). Contigs showing hits only to plant or microorganism sequences were identified as contaminants and deleted from the assembly. The completeness of the assembly was assessed by BUSCO metrics based on evolutionarily informed expectations of housekeeping gene content [26]. Finally, functional annotation was performed using Blast2GO BASIC v4.1 (BioBam, Spain), searching protein domains against the InterPro database [27], GO terms (GOs) from the GO database [28] and metabolic pathways from the KEGG (Kyoto Encyclopedia of Genes and Genomes) database [29].

### *In silico* differential expression analysis

To evaluate the transcriptional responses of *D*. *saccharalis* to each dietary condition, clean reads from cDNA libraries were mapped to our assembly using RSEM software [30]. Transcript abundance estimation and normalization were performed using the fragments per kilobase per million fragments (FPKM) method included in the Trinity package. Expression level and statistical analyses were performed with edgeR software [31]. The quality of replicates was assessed by determining the Pearson correlation between the non-normalized values of the abundance of transcripts in each pair of library replicates. Starvation libraries were excluded from the analysis due to the low correlation coefficients (S1 Fig in S1 File). Thus, the identification of differentially expressed genes (DEGs) was performed by comparing sugarcane-fed larvae to artificial diet-fed larvae. The statistical significance criteria for DEGs were a false discovery rate (FDR) of less than 0.01 and a Log_2_ (fold change) > 2. Finally, GO enrichment analysis was performed using FUNC and REVIGO software [32, 33]. The visualization of expression profiles was performed using HeatMapper and ClustVis [34, 35].

### Phylogenetic analysis

Multiple alignments of the full-length amino acid sequences of genes encoding aminopeptidase N proteins (APNs) was performed using the sequences identified in our transcriptome. Only sequences containing the conserved DEP amino acid sequence motif at the C-terminus of the Cry toxin-binding domain were included, following the selection criteria used in the phylogeny of lepidopteran APNs reported by Hughes in 2014 [36]. Sequences were aligned using the software MAFFT v7 [37]. The phylogenetic tree was constructed with RAxML v8 [38] using the maximum likelihood method with the WAG model, and 1000 bootstrap replicates. The visualization of the phylogeny was performed with the online platform iTOL [39]. The protein sequences used in the phylogenetical analysis are provided in S2 File.

### Reverse Transcription Quantitative real-time PCR (RT-qPCR) validation

The expression of six DEGs related to the transport of toxic substances and candidate receptors for Cry toxins was evaluated by RT-qPCR. The extraction of RNA was performed as described above. For cDNA synthesis, 2 μg of total RNA was treated with DNase I (Invitrogen, Carlsbad, CA, USA), and synthesis of the first-strand cDNA was performed using Oligo(dT) 30 primer. The M-MLV Reverse Transcriptase (Invitrogen, Carlsbad, CA, USA) was used following manufacturer’s instructions. The RT-qPCR was performed in a CFX96 Touch™ Real-Time PCR Detection System (Bio-Rad, Hercules-CA, USA) using the SYBR™ green system (Promega, Madison-Wisconsin, USA). Each reaction was performed with 2 μL of diluted cDNA, the corresponding specific primer pairs at 0.5 μM and 5.0 μL of SYBR™ green in a total volume of 10 μL. In each run, an initial step of 95°C for 15 minutes was followed by 40 cycles of 95°C for 30 seconds and 60°C for 60 seconds. All samples were evaluated in three biological replicates, each from RNA extracted using a pool of 20 larvae. Also, three technical replicates were performed for each RT-qPCR reaction. Primer efficiency was estimated using the software MINER 4.0 [40]. Expression analysis was performed following the Pfaffl method [41] using qbase+ software (Biogazelle). Statistical significance was calculated using REST software [42] with a 0.05 significance level for comparisons between treatments. The stability of six candidate genes obtained from the assembly was assessed using the geNorm algorithm [45], and the genes *β-actin* and *rps10* (ribosomal protein 10) were selected as reference genes. The list of genes evaluated and the primer specifications (designed using PrimerQuest tool, IDT) are provided in S1 Table in S3 File.

## Results

### Sequencing and assembly

In total, 108.2 million PE reads were sequenced from six cDNA libraries of *D*. *saccharalis*. The average library size was 18.3 million PE reads (Table 1). The high-quality PE reads were filtered and further used for digital expression analysis. A *de novo* assembled transcriptome containing 115,346 sequences was generated. From these sequences, 50,206 contigs were identified as putative homologues of proteins found in the NR database of NCBI, of which 27,626 correspond to unigenes (83.9 Mbps). A total of 981 (~0.9%) contigs were identified as contaminant sequences and excluded from the assembly, resulting in 49,225 (42.7%) (Table 2). The raw data was uploaded to the NCBI SRA database under bioproject number PRJNA564321.

**Table 1:** Mean of paired-end reads produced by the sequencing of cDNA libraries from *D*. *saccharalis*.

Samples by Food source	Mean number of raw data PE reads	Mean number of clean PE reads (%)
Sugarcane	22,527,044	21,712,257 (96.4)
Artificial Diet	13,048,639	12,634,615 (96.8)
Starvation	18,529,437	17,919,133 (96.7)

**Table 2:** Trinity statistics for the *D*. *saccharalis* transcriptome.

Feature	Including NA transcripts	Excluding NA transcripts
Number of Unigenes	72,100	27,626
Number of Contigs	115,346	49,225
GC %	36.2	38.62
Completed BUSCOs		1,034 (96.9%)
Single-copy BUSCOs		527 (49.4%)
Duplicated BUSCOs		507 (47.6%)
Median (bp)	655	1188
Mean (bp)	1,158	1,706.08
Total assembled bases	133,579,025	83,982,005
Contig N50 (bp)	1,864	2,633

NA: transcripts without hits in the non-redundant database from NCBI.

GC: guanidine-cytosine content.

### Functional annotation

**Fig 1:**
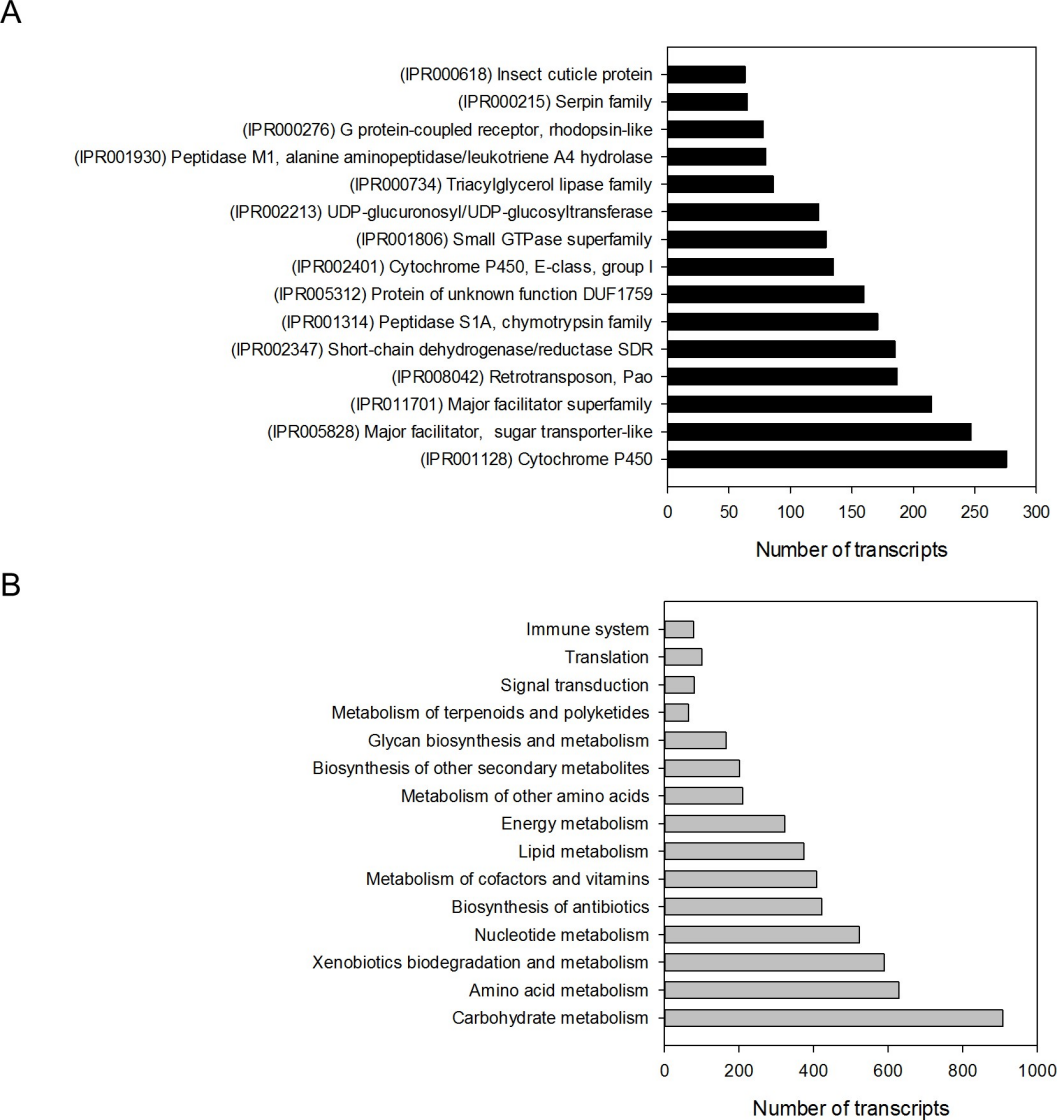
Distribution of annotated transcripts of *D*. *saccharalis* transcriptome assembly. (A) Most common domains, using InterPro database (B) Major metabolic pathways, according to Kyoto Encyclopedia of Genes and Genomes (KEGG) database.

**Fig 2:**
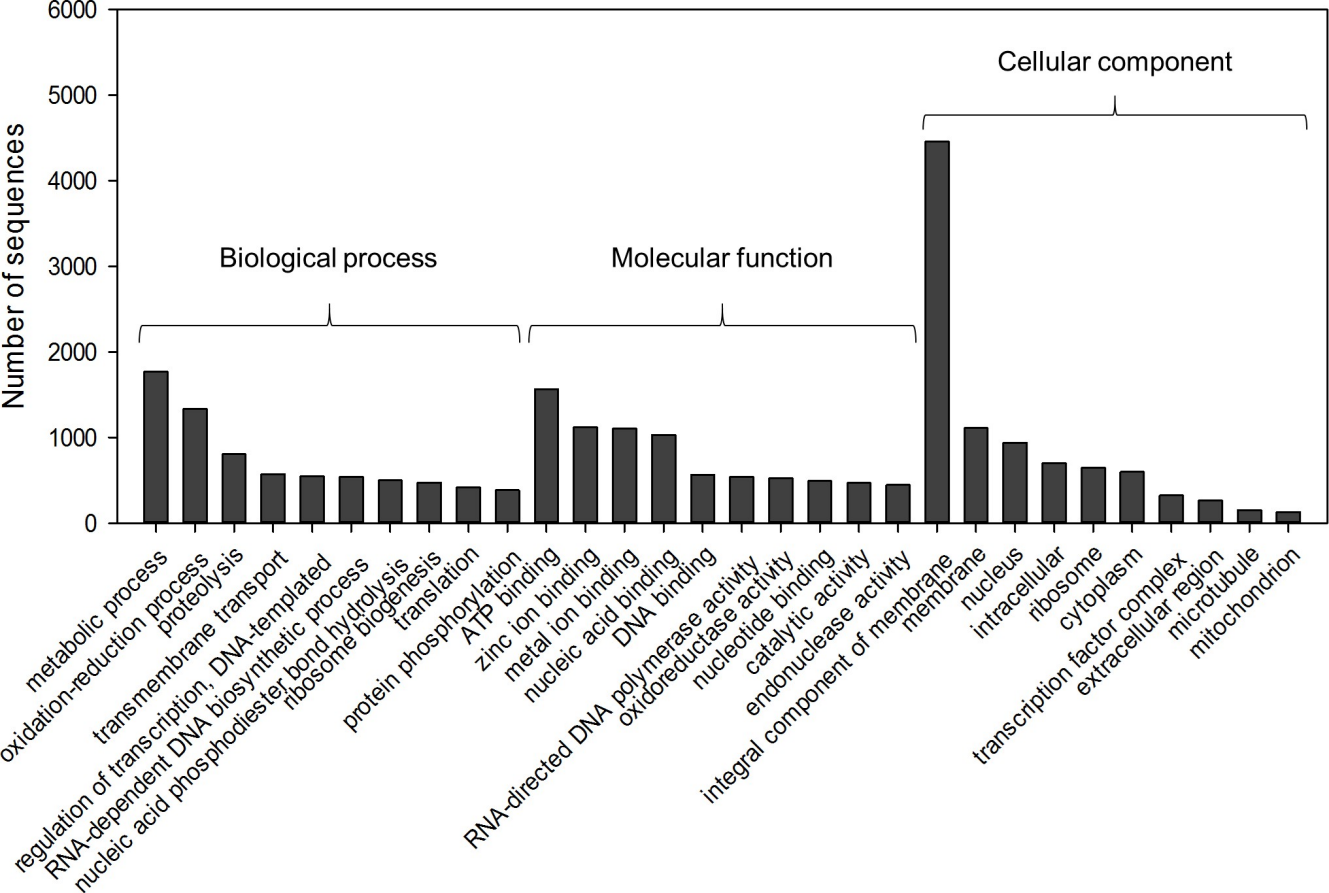
Distribution of sequences for the most abundant GO terms of the three main GO categories.

The *D*. *saccharalis de novo* transcriptome was annotated against the NR, InterPro, GO, Pfam, and KEGG databases to identify the distribution of protein-coding genes (Table 3). The top three species showing hits in the Blast analysis were the lepidopterans *Amyelois transitella*, *Bombyx mori*, and *Papilio xuthus*, with more than 45% matching sequences (S2 Fig in S1 File). InterPro Scan analysis revealed that protein families involved in detox and digestive processes such as cytochrome 450 proteins (CYPs) and major facilitator proteins were the most abundant proteins in the assembly (Fig 1A). The annotation was completed through metabolic pathway analysis, revealing 327 enzymes grouped in 114 metabolic pathways (Fig 1B). Finally, 2,428 GO terms were annotated (Fig 2).

**Table 3:** Number of sequences annotated using each of three main public databases.

Database	Contigs	Unigenes
Gene Ontology	26,584 (54.0%)	15,843 (57.3%)
InterPro Scan	36,330 (73.8%)	20,815 (75.3%)
KEGG	2,077 (4.2%)	1,310 (4.7%)

Percent shown is relative to the total number of transcripts with hit in the non-redundant database from NCBI.

### Gene expression profile and ontology enrichment analysis

**Fig 3:**
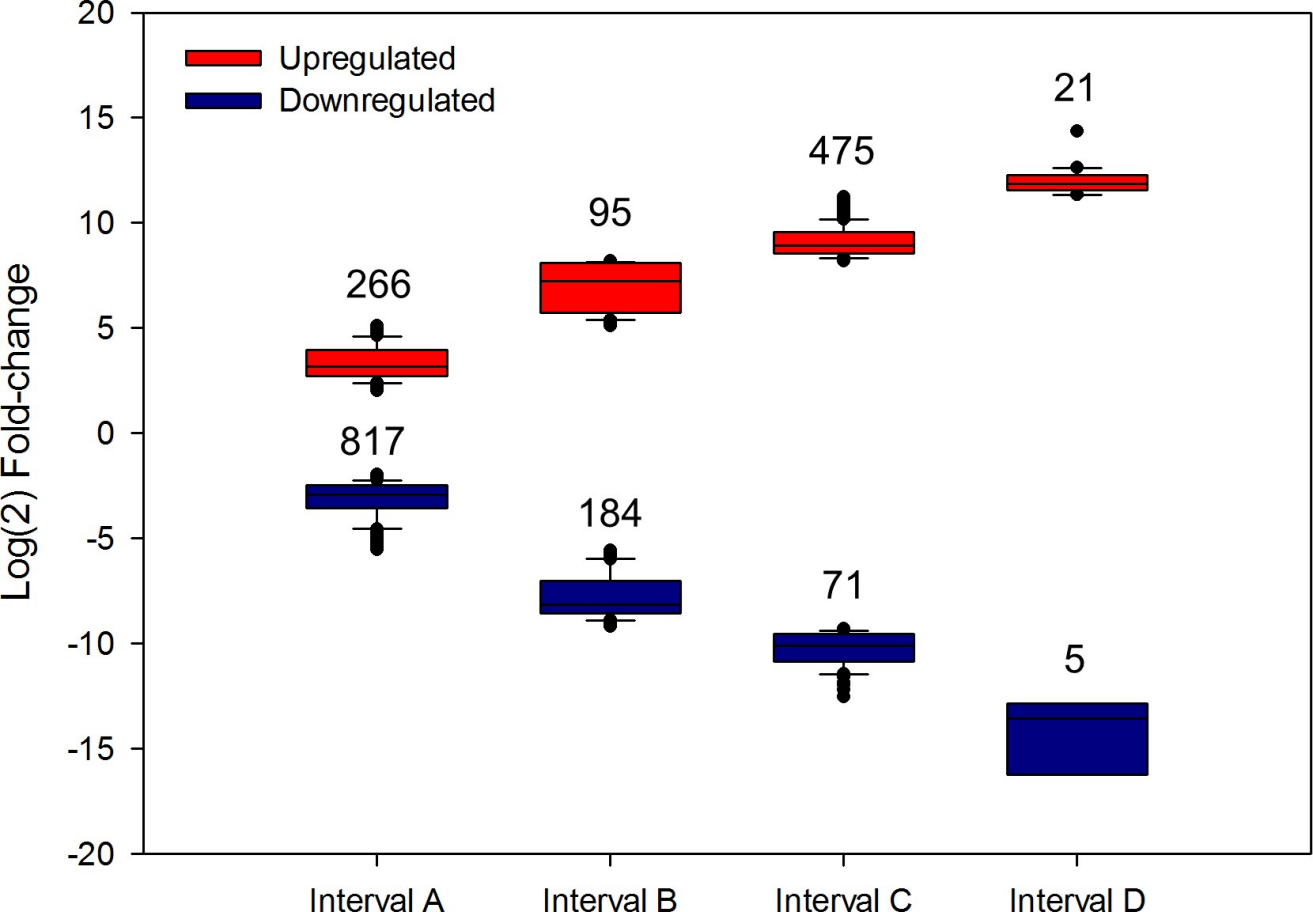
Number of differentially expressed transcripts in sugarcane-fed larvae compared to artificial diet-fed larvae. Upregulated intervals of fold-change correspond to 2 < A < 5.1 < B < 8.2 < C < 11.3 < D < 14. Downregulated intervals of fold-change correspond to -2 > A > -5.6 > B > -9.2 > C > -12.8 < D < 16. Total number of transcripts in each interval is shown above boxes. Expression values are represented as Log (2) fold-change.

The transcripts from each cDNA library were normalized via the FPKM method. For this purpose, an average of 83.2% of the clean reads was successfully mapped against our assembly. A total of 1,934 transcripts (1,697 unigenes) were identified as differentially expressed, among which 857 and 1,077 transcripts were upregulated and downregulated, respectively, in sugarcane-fed larvae, in comparison to artificial diet-fed larvae. In Fig 3, we show the distribution of transcripts across different fold-change intervals (each interval representing 1/8 of the total range of variation). This was made to describe how the intensity of the regulation, represented by the fold-change, is distributed in our analysis. Thus, we can observe that most of the genes present a slight regulation of gene expression (interval A), while just a few transcripts are strongly regulated (interval D) in response to diet sources. A heatmap showing the overall expression profiles is provided in S3 Fig in S1 File.

**Fig 4:**
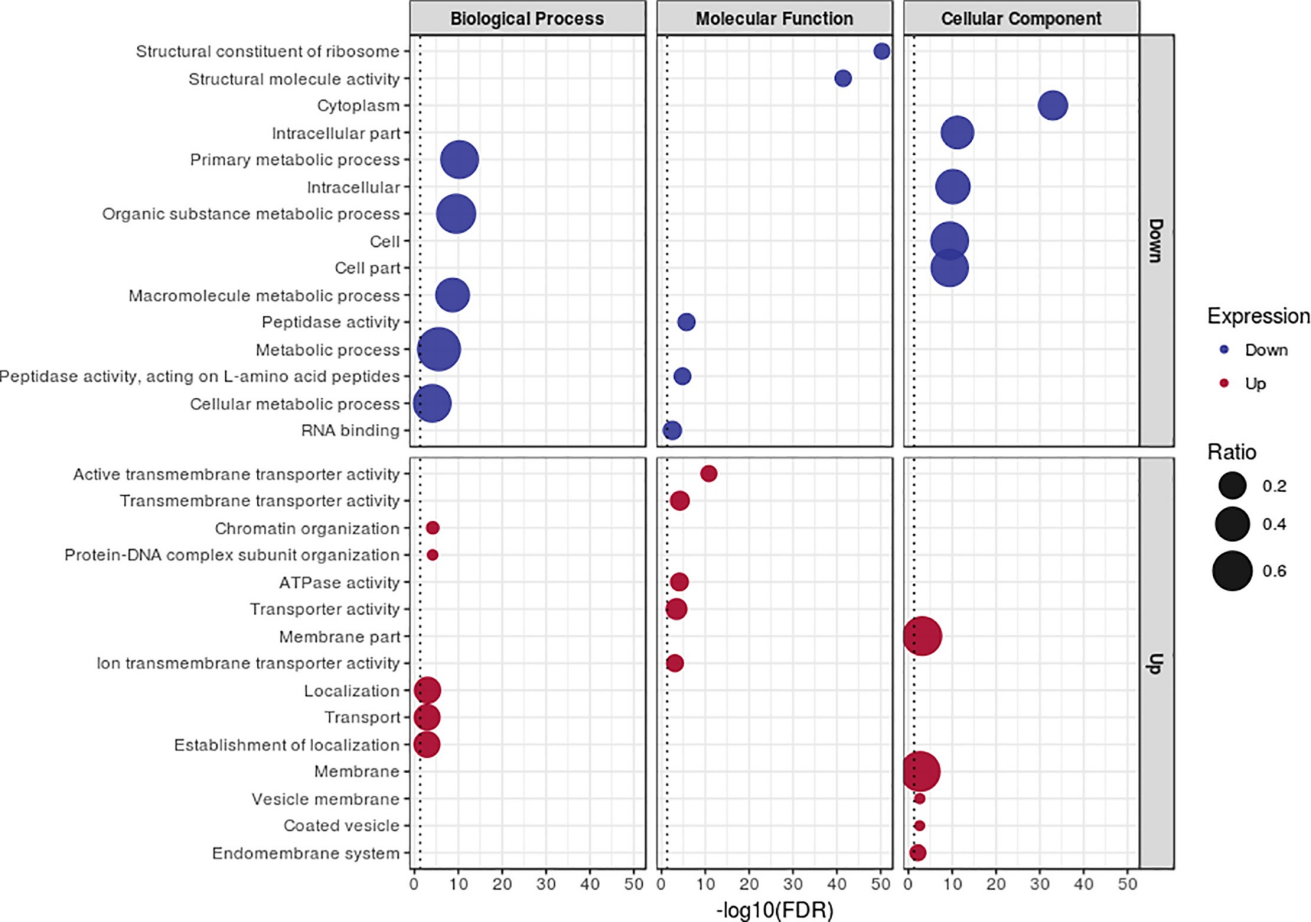
Frequency distribution of the main enriched GO terms for each DEG dataset. Statistical significance is represented by the size of the circles. The GO terms shown were considered in a multilevel distribution. The regulation of gene expression is shown for sugarcane-fed larvae in comparison to artificial diet-fed larvae.

To provide a functional overview of the transcriptional response to food sources, GO enrichment was performed separately for each DEG dataset. A total of 244 GO terms were obtained. The most frequent GO categories for each set of DEGs are shown in Fig 4 (full list provided in S2 Table in S3 File). Genes annotated with GO terms related to the transport of different molecules were observed mainly among the transcripts that were upregulated in sugarcane-fed larvae. For downregulated genes of the same condition, the GO terms with higher frequencies were related to catabolic functions, such as protein metabolism.

### Digestion, detoxification and nutrient transport are the main processes affected by diet

**Fig 5:**
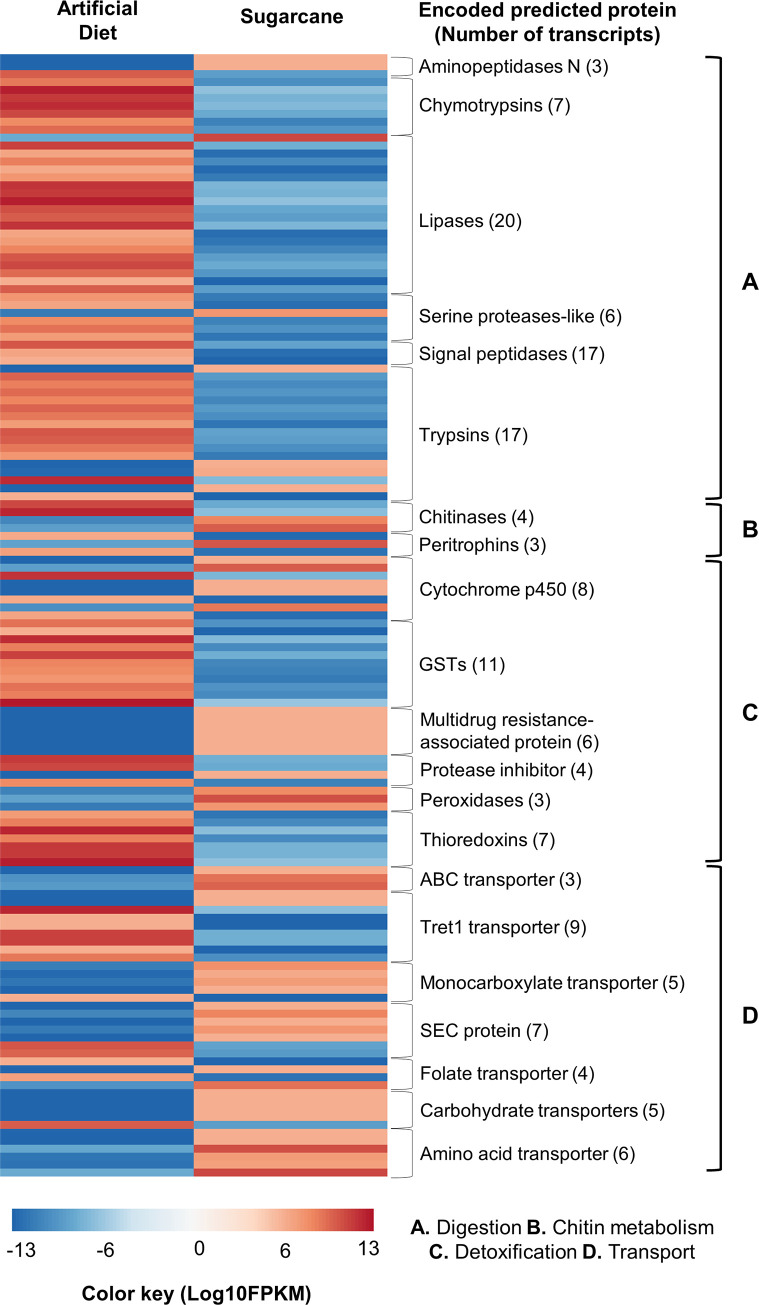
Expression profile of highlighted DEGs in the gut transcriptome of *D*. *saccharalis*. Major metabolic processes affected by the food source are shown. A. Defence. B. Chitin metabolism. C. Detoxification. D. Digestion. E. JH metabolism. F. Signaling. G. Transport. FPKM = Fragments per kb per million fragments. Statistical significance of differentially expressed genes is determined by a false discovery rate (FDR) < 0.01 and a Log2 (fold change) > 2.

Genes encoding proteins involved in processes such as digestion, detoxification, transport, and chitin metabolism showed modulation of their expression by the diet source (Fig 5). Most of the transcripts were related to detoxification and digestion functions. Genes encoding glutathione s-transferases (GSTs) e thioredoxins were mainly downregulated in the sugarcane-fed insects. Otherwise, peroxidases and multidrug resistance-associated proteins were overexpressed. Also, genes encoding digestive enzymes, mainly involved with protein catabolism, were mostly overexpressed in larvae fed on an artificial diet. Differently, most of the genes annotated with the GO term “molecule transport” were overexpressed in sugarcane-fed larvae, with the facilitated trehalose transporter TRET1 and the SEC translocon mediated protein as the predicted proteins with most numbers of transcripts overexpressed.

### Candidate APN genes encoding receptors of Cry toxins

**Fig 6:**
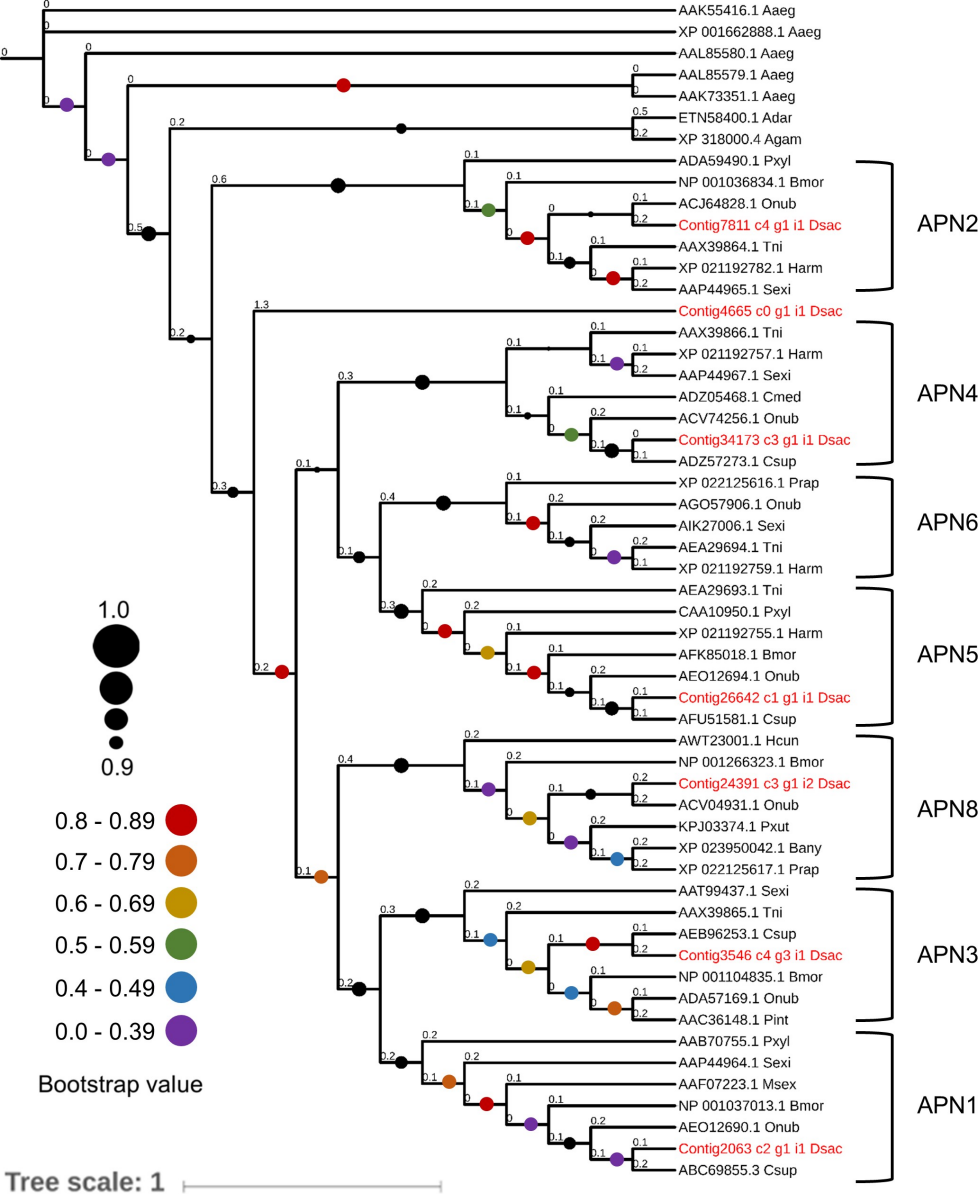
Phylogenetic classification of full-length Lepidoptera APN amino acid sequences. Sequences reported in GenBank and APN contig sequences obtained from the gut transcriptome of *D*. *saccharalis* (highlighted in red) were used. The accession numbers of the amino acid sequences are indicated to the left of abbreviated species names. Dsac: *D*. *saccharalis*, Adar: *Anopheles darlingi*, Agam: *Anopheles gambiae*, Aaeg: *Aedes aegypti*, Sexi: *Spodoptera exigua*, Harm: *Helicoverpa armigera*, Tni: *Trichoplusia ni*, Pxyl: *Plutella xylostella*, Bmor: *B*. *mori*, Onub: *Ostrinia nubilalis*, Pint: *Plodia interpunctella*, Csup: *Chilo suppressalis*, Msex: *M*. *sexta*, Hcun: *Hyphantria cunea*, Prap: *Pieris rapae*, Bany: *Bicyclus anynana*, Cmed: *Cnaphalocrocis medinalis*. Bootstrap values over 0.9 are represented by black circles. Bootstrap values for less robust clades are represented by colorful circles. Branch lengths are given at the start of each branch.

All seven full-length APN sequences analysed contained the conserved DEP amino acid motif within the Cry toxin-binding domain [43] and the two conserved GA(X)_1_EN and HEXXH(X)_18_E motifs (S4 Fig in S1 File). The classes APN1, APN2, APN3, APN4, APN5, and APN8 were identified through our phylogenetic analysis according to the lepidopteran APN classification proposed by Crava *et al*. 2010 [44] (Fig 6).

### Validation of RNA-Seq data by RT-qPCR

**Fig 7:**
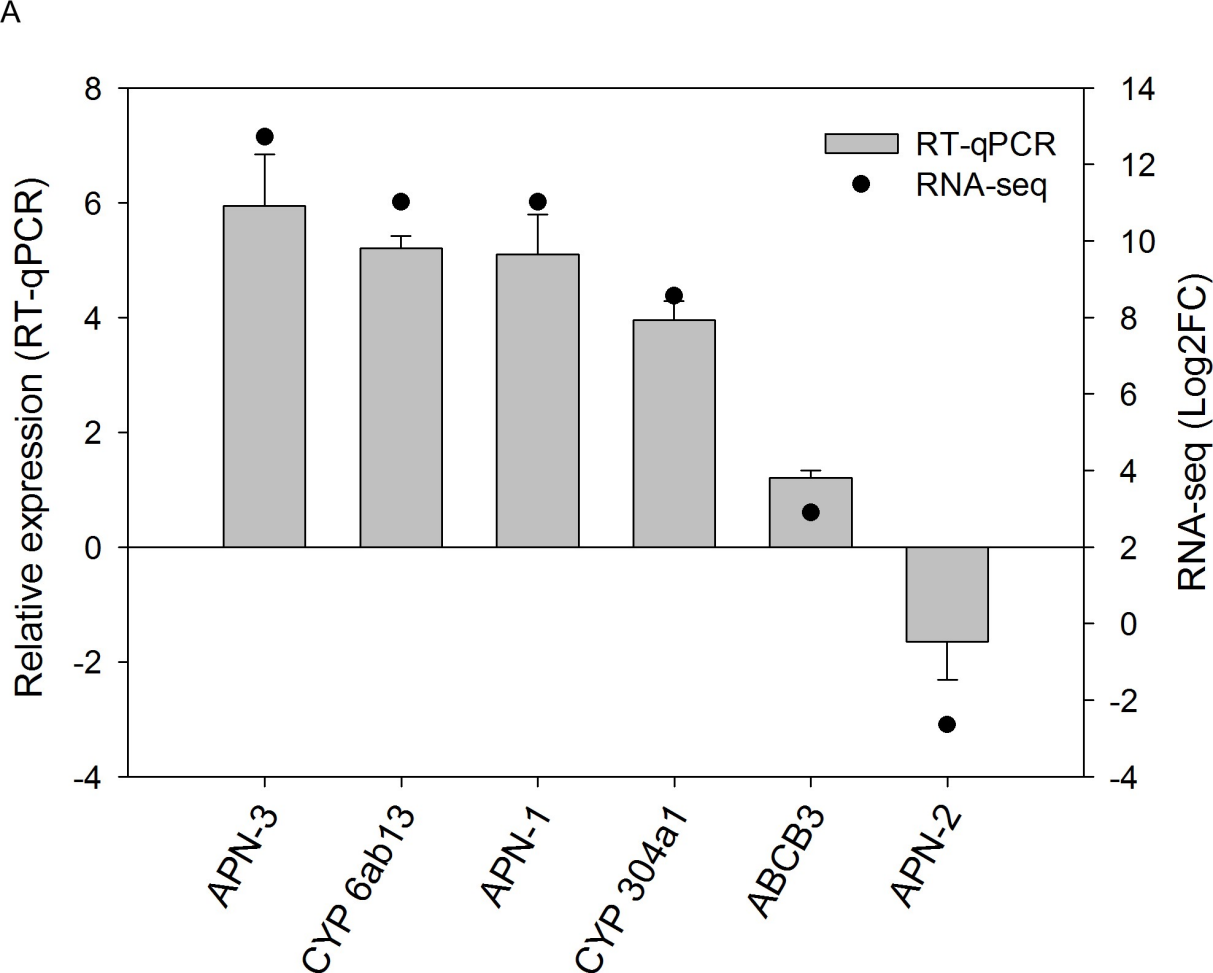
Expression patterns of differentially expressed *D*. *saccharalis* genes validated by RT-qPCR. *cyp*: cytochrome P450 protein. *abc*: ABC transporter. *apn*: aminopeptidase N.

The genes encoding the CYP proteins 6ab13 and 304a1, the ABC transporter ABCB3 and three aminopeptidase N proteins (APN-1, APN-2, and APN-3) showed a high correlation (>0.9) with the RNA-Seq data (Fig 7). The results of geNorm analysis to assay stability of the reference genes are given in S5 Fig in S1 File. The genes selected as reference genes were *actin* and *rsp10* (ribosomal protein 10), which presented the smaller M value (average expression stability). As recommended by Hellemans & Vandesompele [45], optimal reference genes must present M < 0.5, indicating a higher suitability.

## Discussion

Here, we identified 27,626 protein-coding unigenes in the gut transcriptome of *D*. *saccharalis*. Tissue-specific transcriptomes of other lepidopterans have shown that despite the high variability in contig numbers, the number of protein-coding unigenes is consistently between 20,000 and 30,000 [9, 12, 46–48]. Insect transcriptomes have been sequenced at higher frequency in the last five years, proving to be useful as a descriptive tool and an invaluable source of sequences for studies researching gene function and design of RNAi experiments [2, 10, 13, 49, 50]. In this study, we generated a tissue-specific transcriptome of the sugarcane borer’s gut. The number of transcripts in our assembly (115,346) was higher than the 84,678 transcripts reported in the previous transcriptome [21]. However, 66.5% of the transcripts in the current study were not annotated. The presence of artificial sequences generated in the assembly along with non-coding RNAs and genes of unknown function is the most feasible explanation for this. Here, we were focused on the study expression of protein-coding genes known as blast hits.

Diet is one of the main factors that influence physiology and development in insects, especially in the larval stages of holometabolous insects [2]. The transcriptional responses of different insect species fed on natural and artificial diets have been described by comparing their mRNA profiles [9, 51, 52]. These studies emphasize that detoxification and nutrition are the processes that exhibit higher variation, influenced by different food sources. Furthermore, it was demonstrated that natural diets induce genes that may correspond to a large gut remodeling by physiological adaptation. Following, we discuss the main groups of genes found in our study.

### Molecular transport

The transport of molecules in the insect gut is associated mainly with nutrition and detoxification [7, 53]. Midgut cells show a high expression of genes encoding transport proteins involved in these processes [2]. In our work, this group of genes was upregulated in larvae fed on sugarcane. Based on the results reported by Roy *et al*. [9] in two species of the genus *Spodoptera*, a strong hypothesis suggesting that specialist insects display more diet-specific responses than generalists could explain the profiles of some genes identified in our data. As discussed by Vogel *et al*. [2] specialists require efficient transport to obtain the maximum amount of the limited nutrients offered by a narrow range of hosts. Additionally, Govind *et al*. [54] showed that the specialist lepidopteran *Manduca sexta* responds to a nicotine-rich diet by upregulating ABC-transporters coding genes, suggesting a detox role of these genes. Different types of transporter molecules were detected among our set of DEGs (Fig 5). We found that 40 of the predicted proteins from DEGs overexpressed in sugarcane-fed larvae are transmembrane transporters. It is expected to observe high expression levels of these genes in the gut, due to the active absorption rates in this tissue. Moreover, the expression profile found in this study could be related to a need for a more efficient absorption process in a restrained diet, as the sugarcane thatch compared to any artificial diet.

Also, is important to highlight the presence of ABC transporters, which are proteins of great interest for biotechnological applications, in our work expression of predicted ABC transporters was higher in insects fed with sugarcane. Genes from this family have been reported to play a role in the development of resistance to insecticides and even adaptation to plant defences, mainly by eliminating molecules through the gut membranes [55, 56]. Furthermore, some ABC transporters act as receptors for *Bt* toxins [57, 58]. Thus, this class of genes can be useful as targets for the design of strategies based on RNAi for the management of the sugarcane borer.

### Digestive enzymes

Major digestion steps in insects are performed by catalytic enzymes produced by the gut cells [7]. It has been demonstrated that the expression levels of genes encoding digestive enzymes are variable depending on the food source and the degree of insect specialization [46, 59–61]. Our enrichment analysis elucidated several major digestive processes, such as the metabolism of lipids and proteins, which were overexpressed in larvae fed on an artificial diet. As observed in Fig 4, almost all digestion-related genes, except for those encoding aminopeptidases, were overexpressed in larvae fed with artificial diet. In accordance with other studies [9, 59, 61], these results are expected in specialists due to the increasing availability of nutrients in artificial diets in comparison to natural hosts. The large number of identified genes encoding serine proteases, such as trypsin and chymotrypsin, probably indicated a response to the presence of proteinase inhibitors in the diet. These molecules induce the expression of many isoforms of serine protease genes in many insects [2, 46, 59]. Notably, genes associated with carbohydrate metabolism were not significant among the DEGs. As sugarcane is rich in carbohydrates, no significant nutritional differences are expected for these macromolecules between artificial diet and sugarcane.

### Detoxification

Genes related to detoxification are strongly regulated in insects that are fed on different diets. The gut is the first defence barrier against pathogens and toxins acquired through food [2, 62]. Well-characterized families of enzymes that play crucial roles in detox pathways, such as GST, UGT, and CYP proteins and peroxidases, were identified as DEGs here and in previous studies on Lepidoptera [9, 12, 46, 61, 63, 64]. Most of these gene families have variable expression profiles; some transcripts are overexpressed in sugarcane-fed larvae and others overexpressed in artificial diet-fed larvae, probably due to the high structural and functional diversity of these families (Fig 5). However, GSTs and thioredoxins are all upregulated in artificial diet-fed larvae, while multidrug resistance-associated proteins are down-regulated in the same condition.

As suggested in recent publications [12, 46], increasing expression of GST and CYP coding genes could be one of the initial steps in specialist adaptation to different diets. The transition to new food sources is challenging for specialists because their tolerance to high doses of non-specific toxic compounds is deficient [5]. Artificial diets are usually composed of different vegetal components and may include toxins and inhibitors [22]. Since the strain of *D*. *saccharalis* used in this work was raised on an artificial diet, and overexpressed set of genes involved in detox processes was expected to exist in the larvae fed with artificial diet.

### Chitin metabolism

The metabolism of chitin in the gut is a critical process for insect survival. As the first barrier in the gut, the peritrophic membrane (PM) is continuously exposed to damage. Therefore, the remodeling of this tissue is essential to maintain the gut's normal functions [8]. Since chitin is one of the main molecules in the PM composition, enzymes belonging to chitin degradation and biosynthesis pathways need to be produced to maintain the integrity of the chitin matrix. Our results showed that genes encoding some of these enzymes were upregulated in sugarcane-fed larvae (Fig 5). Genes involved in chitin metabolism have been studied recently as promising targets for RNAi [65–67]. The interruption of PM remodeling in *D*. *saccharalis* via RNAi targeting the genes identified in this study could be a potential alternative for sugarcane borer management. Further, it has been demonstrated in some species that even a permeabilized PM is not lethal for the insect, and no direct effect on nutrition is observed. However, it is important to emphasize that the combined use of toxic compounds with permeabilized PMs generally leads to deleterious phenotypes and mortality [68–70]. Thus, RNA interference from genes involved with PM remodeling could increase pests' susceptibility to specific insecticides, leading to a reduction in the doses of pesticides delivered in the field.

### Phylogenetic analysis of APNs

The *B*. *thuringiensis* crops have been used to control infestations of lepidopteran pests in a wide range of hosts for over fifteen years. However, the resistance becomes a significant challenge for this technology in recent years, mainly because of the variability in Cry receptors present in the gut of most insects [71]. For example, the multigenic APN family is a complex of highly conserved proteins that can act as receptors for Cry toxins in most insects and regulate the toxicity of the first. Amino acid sequence changes in specific motifs of APNs, such as the binding domain, contribute to reducing affinity to Cry toxins [43]. Thus, the evaluation of the class-specific affinity of APNs to Cry toxins is useful for improving *Bt* technology [36]. Moreover, the evolution of the APN family in Lepidoptera is assumed to be closely related to selective pressure associated with their enzymatic function and the environmental presence of Cry toxins [36, 44].

Herein, we were able to classify some APNs present in the gut of *D*. *saccharalis*. In addition to the three previously characterized isozymes of *D*. *saccharalis* [72], three new APNs belonging to three different classes were identified. However, for a better understanding of the interactions between Cry toxins and APNs in Lepidoptera, it will be important to perform the functional characterization of some APN sequences found in this study. Successful attempts to identify the specificity of APN receptors to certain Cry toxins have been performed in the past, laying the basis for their biotechnological application [73–75].

## Conclusions

All the data presented here contributed to characterize the gut transcriptome of *D*. *saccharalis*, which will serve as a basis for future research on *D*. *saccharalis* genomics, and as a source of annotated genes that could be targeted for pest management by using RNAi. Furthermore, details of the larvae molecular physiology involved in response to natural and artificial diets were revealed through differential gene expression analysis. The identification of the protein repertoire of the sugarcane borer and its ability to modulate gene expression involved in certain pathways and processes could be the key to understanding the adaptation to new diets that occur in specialist insects.

## Supporting information

S1 File (PDF)

S2 File Amino acid sequences of APNs used in the phylogenetic tree reconstruction. (FAS)

S3 File (PDF)
