# Supplementary material for: Comparative gut transcriptome analysis of Diatraea saccharalis in response to the dietary source
Source: PLoS One. 2020 Aug 3;15(8):e0235575. doi: 10.1371/journal.pone.0235575 (PMC7398519; doi:10.1371/journal.pone.0235575)
Supplement: S1 File — (PDF) [file pone.0235575.s001.pdf]

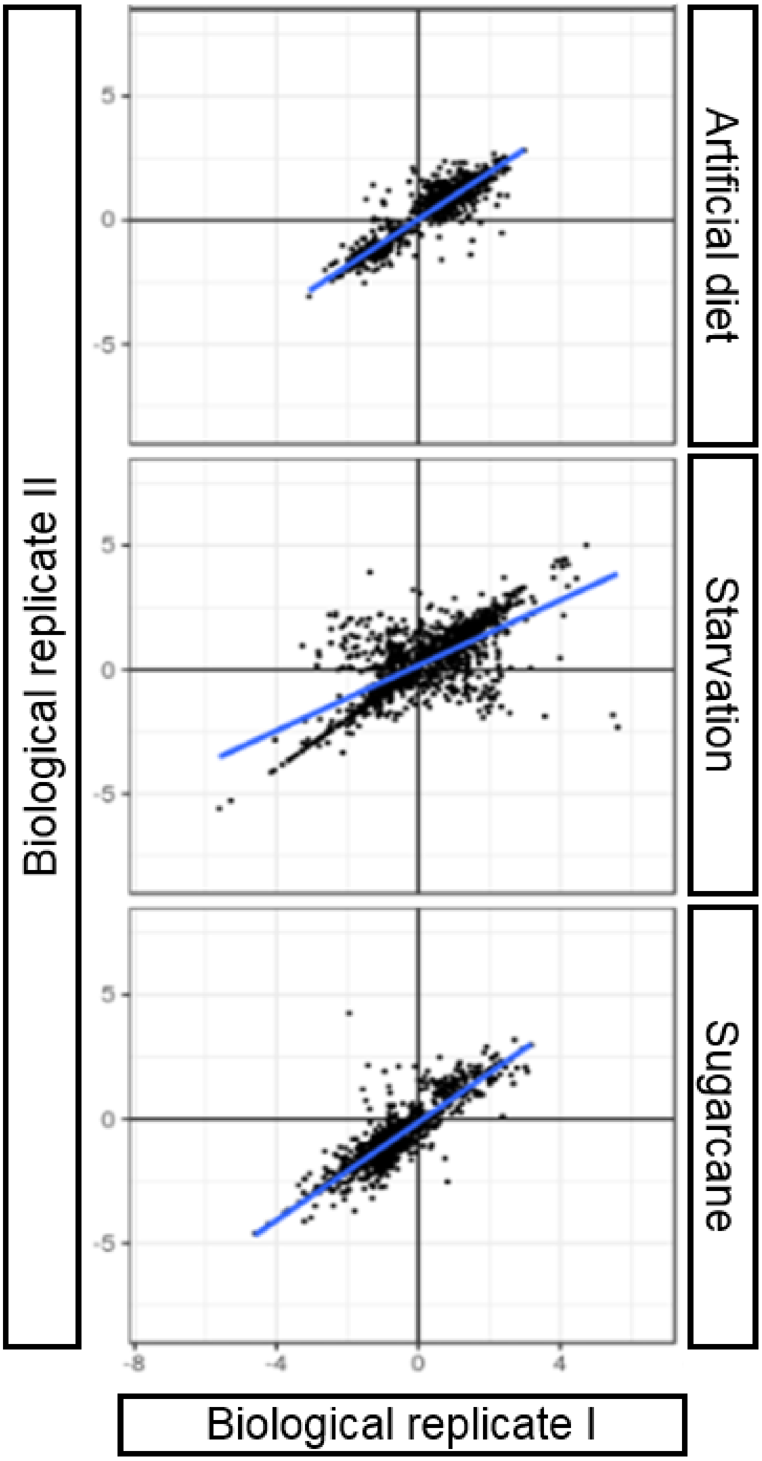

Fig A. Pearson correlation between replicates obtained from Illumina sequencing.

Correlation values correspond to 0.95, 0.44 and 0.89 for artificial diet, starvation and sugarcane treatments, respectively.

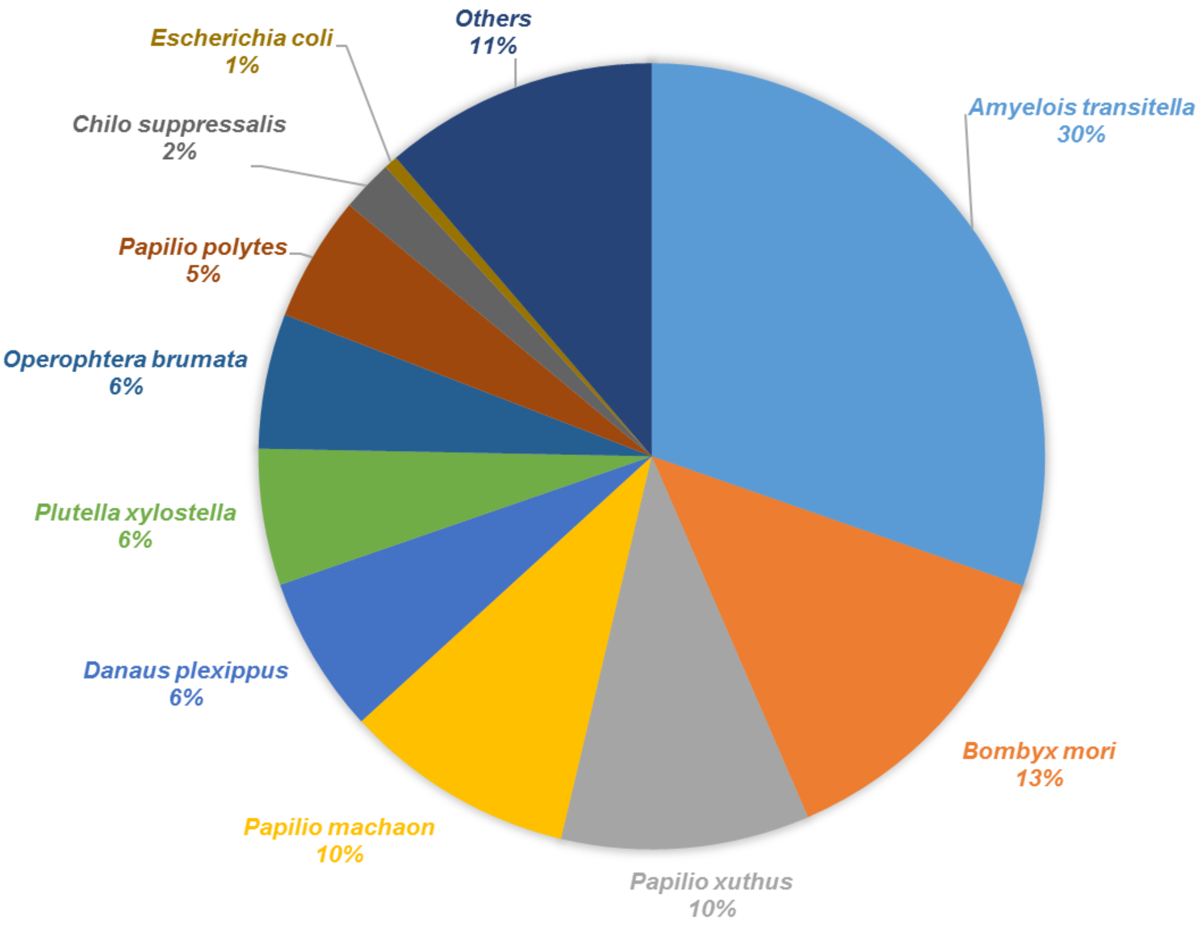

Fig B. Top-hit species distribution from blast analysis against protein NR database of the NCBI.

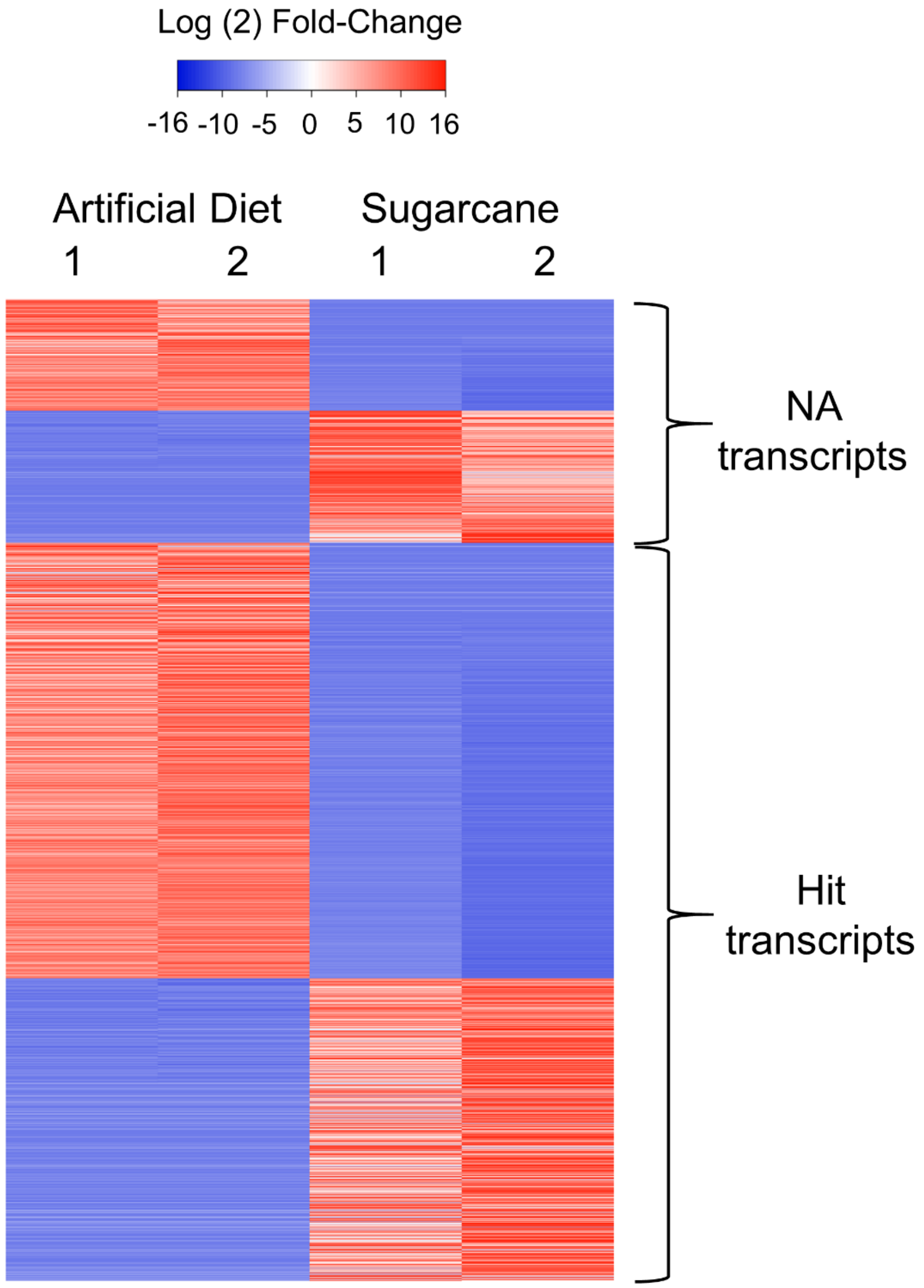

Fig C. Heatmap of the main clusters generated by edgeR following differential expression analysis of *D. saccharalis* RNA-seq libraries.

Numbers represent sequencing replicates. Fold-change is calculated based on FPKM (fragments per kilobase per million of reads) values for each transcript. NA transcripts: withouth hits in the non-redundant database from NCBI. Hit transcripts: with at least one hit in the non-redundant database from NCBI.

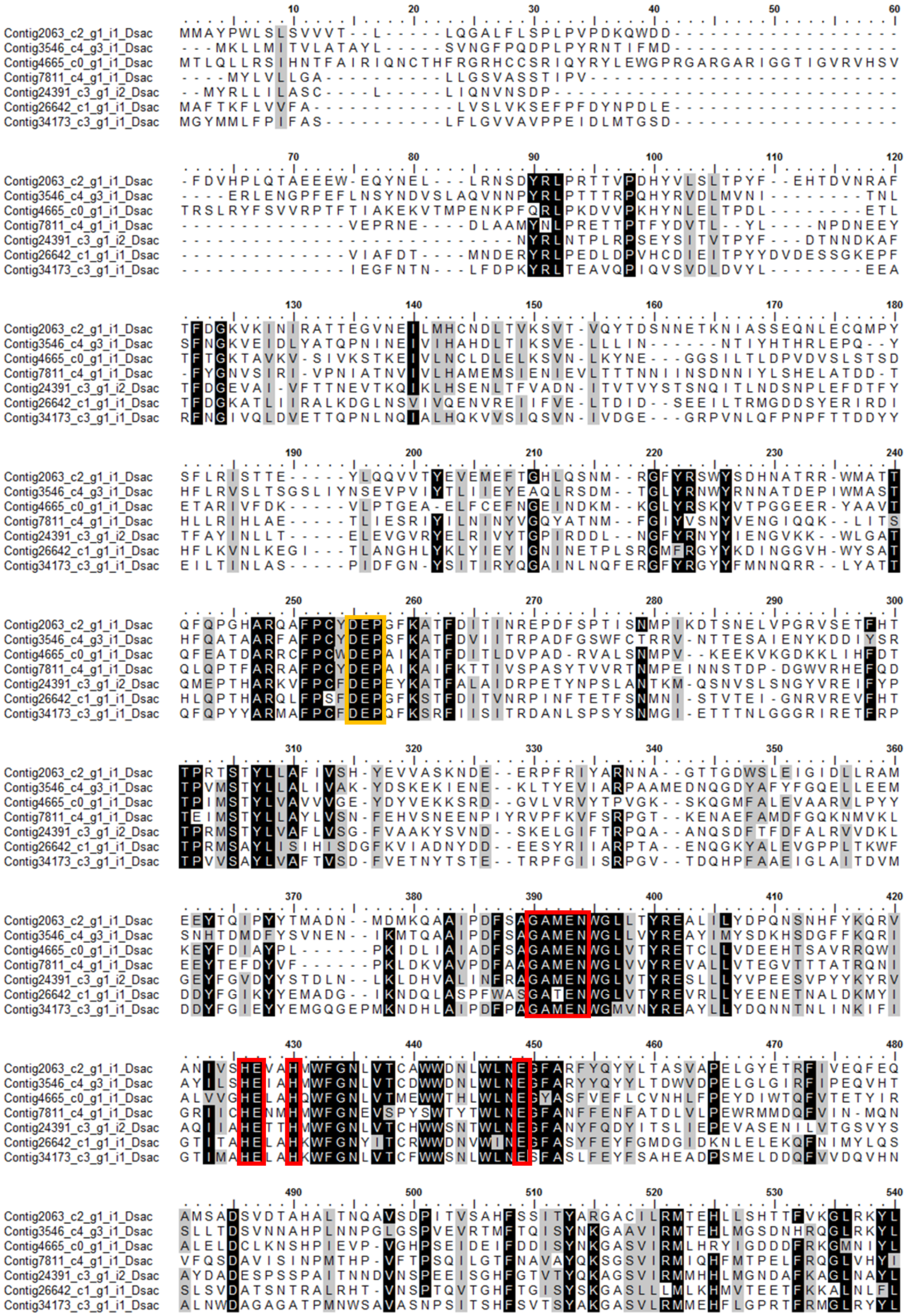

Figure continues in the next page

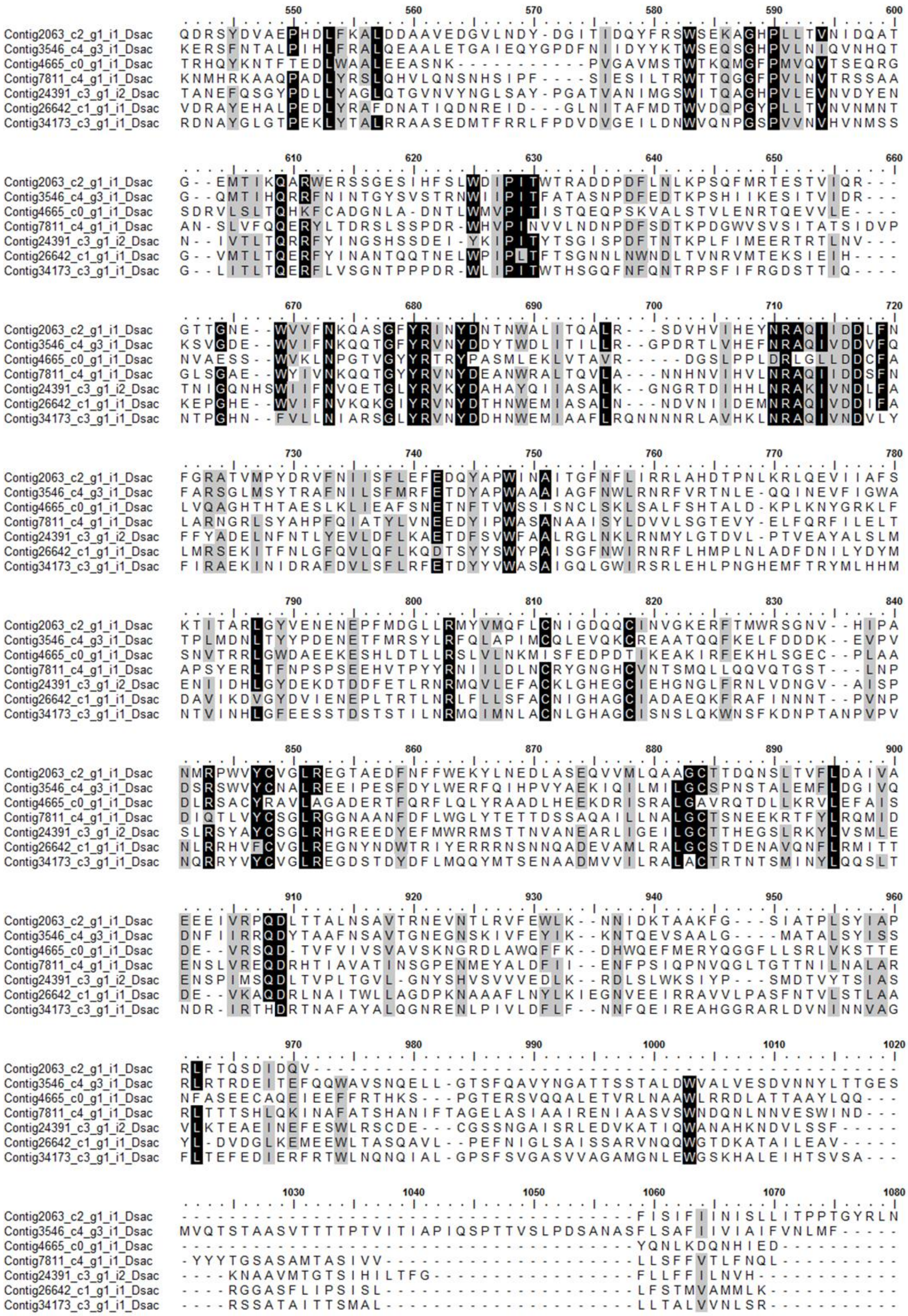

Fig D. Alignment of the seven APNs predicted proteins obtained from the midgut transcriptome of *D. saccharalis*.

Yellow box correspond to the Cry toxin-binding domain and the red boxes to the two conserved motifs: GA(X)1EN and HEXXH(X)18E.

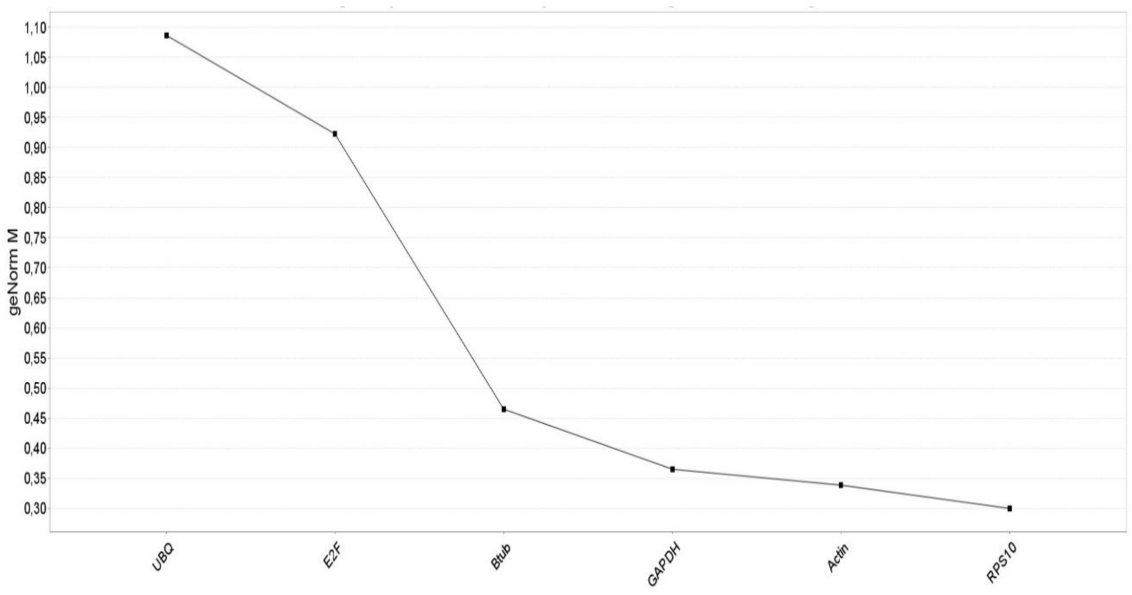

Fig. E. Average expression stability values (M) of candidate reference genes.

Samples used were obtained from *D. saccharalis* larvae treated with different feeding conditions (sugarcane thatch, artificial diet and starvation).
