# Supplementary material for: Comparative gut transcriptome analysis of Diatraea saccharalis in response to the dietary source
Source: PLoS One. 2020 Aug 3;15(8):e0235575. doi: 10.1371/journal.pone.0235575 (PMC7398519; doi:10.1371/journal.pone.0235575)
Supplement: S3 File — (PDF) [file pone.0235575.s003.pdf]

Gene	Primer Sequence Forward (5'-3')	Primer Sequence Reverse (5'-3')	Amplicon (bp)	Efficiency (MINER)
**β-actin**	CTTGGGTATGGAAGCTAACG	TCTTGATCTTCATGGTTGATGG	188	1.95
**rsp10**	TGATCGCTTCAGTGTTGCTG	TCTCAAATCCAGGGGTTACG	160	1.93
**ubq**	TCTACACTACATCTTGTCTTGC	GGATACCTTCCTTGTCTTGG	145	1.89
**e2f**	CGACTTCAGGAATCAAACGG	AGAGGAACACTTCTATCTCACC	131	1.91
**Btub**	CAAGAACTCGTCCTACTTCGTC	CGGTGAACTGTTCCGAGATG	155	1.88
**gapdh**	CATCAAGCAGAAGGTCAAGG	GTTGTCATTGAGTGAGATACCG	148	1.88
**cyp_6ab13**	TATTTCCGTGAACCGGAGAAG	GCTATGCCAGCCATTGATTG	152	1.91
**cyp_304a1**	GCTCTCCACATGGACAAAGAG	CATAGTTTGCCGCGCATAAG	159	1.91
**ABCB3**	GGTTCTACGACCCTGATGTTG	TTGTCACCGTACGCGATATTC	157	1.90
**APN-1**	TCGTGAAGGAACAGCTGAAG	TTCCTCCTCCGCAACAATAG	157	1.89
**APN-2**	GGACCTCTCTCTGTGGAAATC	CCTGCTGATTGCACCATTAC	154	1.90
**APN-3**	TCCGCTATCAAGGTGCAATC	GCTCATCAAAGCATGGGAAAG	147	1.90

Table A. List of genes used for RT-qPCR validation and primers specifications.

β-actin: beta actin. rsp10: ribosomal protein 10. ubq: ubiquitin. e2f: elongation factor e2f. Btub: beta tubulina. gapdh: glyceraldehyde 3-phosphate dehydrogenase. cyp_6ab13: cytochrome p450 6ab13. cyp_304a1: cytochrome p4503 04a1. ABCB3: ATP-binding cassete family B3. APN-1: aminopeptidase N class 1. APN-2: Aminopeptidase N class 8. APN-3: aminopeptidase class 4.

**Upregulated**			
**GO term (name)**	**GO term (id)**	**P-value**	**Frequency**
active ion transmembrane transporter activity	GO:0022853	0.002436	0.92%
active transmembrane transporter activity	GO:0022804	1.54E-11	2.83%
ATPase activity	GO:0016887	7.87E-05	4.56%
ATPase activity, coupled	GO:0042623	1.56E-06	2.50%
ATPase activity, coupled to movement of substances	GO:0043492	3.13E-08	1.59%
ATPase activity, coupled to transmembrane movement of substances	GO:0042626	3.13E-08	1.54%
ATPase coupled ion transmembrane transporter activity	GO:0042625	0.002436	0.85%
beta-catenin binding	GO:0008013	0.001161	0.02%
cation transmembrane transporter activity	GO:0008324	0.002093	2.43%
cation-transporting ATPase activity	GO:0019829	0.002436	0.61%
chromatin assembly	GO:0031497	4.57E-05	0.10%
chromatin assembly or disassembly	GO:0006333	5.82E-05	0.12%
chromatin organization	GO:0006325	6.78E-05	0.67%

coated vesicle	GO:0030135	0.002668	0.21%
coated vesicle membrane	GO:0030662	0.002668	0.15%
COPII vesicle coat	GO:0030127	0.001692	0.05%
COPII-coated ER to Golgi transport vesicle	GO:0030134	0.001692	0.21%
cytoplasmic vesicle membrane	GO:0030659	0.002668	0.23%
DNA packaging	GO:0006323	5.82E-05	2.81%
endomembrane system	GO:0012505	0.006866	0.07%
ER to Golgi transport vesicle membrane	GO:0012507	0.001692	0.05%
establishment of localization	GO:0051234	0.001416	17.76%
Golgi-associated vesicle	GO:0005798	0.002668	0.10%
Golgi-associated vesicle membrane	GO:0030660	0.002668	0.09%
inorganic cation transmembrane transporter activity	GO:0022890	0.001252	1.90%
inorganic molecular entity transmembrane transporter activity	GO:0015318	0.000971	0.00%
ion transmembrane transporter activity	GO:0015075	0.000826	3.73%
localization	GO:0051179	0.001054	18.50%
membrane	GO:0016020	0.002079	61.59%
membrane part	GO:0044425	0.000705	57.39%
metal ion transmembrane transporter activity	GO:0046873	0.001163	1.00%
monovalent inorganic cation transmembrane transporter activity	GO:0015077	0.001281	1.39%
neurotransmitter transport	GO:0006836	0.000211	0.11%
neurotransmitter transporter activity	GO:0005326	0.000127	0.06%
neurotransmitter:sodium symporter activity	GO:0005328	0.000127	0.06%
nucleosome assembly	GO:0006334	4.57E-05	0.09%
nucleosome organization	GO:0034728	4.57E-05	0.13%
P-P-bond-hydrolysis-driven transmembrane transporter activity	GO:0015405	3.13E-08	1.69%
primary active transmembrane transporter activity	GO:0015399	3.13E-08	1.69%
protein-DNA complex assembly	GO:0065004	7.32E-05	0.20%
protein-DNA complex subunit organization	GO:0071824	7.32E-05	0.24%
secondary active transmembrane transporter activity	GO:0015291	0.000127	0.95%
sodium ion transmembrane transporter activity	GO:0015081	0.000598	0.22%
solute:cation symporter activity	GO:0015294	3.67E-05	0.18%
solute:sodium symporter activity	GO:0015370	0.000127	0.12%
symporter activity	GO:0015293	4.11E-05	0.29%
transmembrane transporter activity	GO:0022857	6.27E-05	5.87%

transport	GO:0006810	0.001308	17.62%
transport vesicle	GO:0030133	0.001814	0.18%
transport vesicle membrane	GO:0030658	0.001364	0.10%
transporter activity	GO:0005215	0.000373	8.49%
vesicle coat	GO:0030120	0.002668	0.13%
vesicle membrane	GO:0012506	0.002668	0.22%
**Downregulated**			
amide biosynthetic process	GO:0043604	6.72E-42	6.37%
arginine biosynthetic process	GO:0006526	0.004499	0.31%
arginine metabolic process	GO:0006525	0.004499	0.39%
argininosuccinate synthase activity	GO:0004055	0.002987	0.04%
ATP biosynthetic process	GO:0006754	2.82E-05	0.43%
ATP metabolic process	GO:0046034	5.06E-06	1.26%
ATP synthesis coupled electron transport	GO:0042773	0.000207	0.22%
ATP synthesis coupled proton transport	GO:0015986	2.82E-05	0.41%
biosynthetic process	GO:0009058	2.87E-21	31.61%
carbohydrate derivative biosynthetic process	GO:1901137	0.010125	3.65%
catalytic complex	GO:1902494	0.001644	3.73%
cation transmembrane transport	GO:0098655	0.005418	2.29%
cell	GO:0005623	4.06E-10	53.55%
cell part	GO:0044464	4.06E-10	52.39%
cellular amide metabolic process	GO:0043603	3.77E-43	6.88%
cellular biosynthetic process	GO:0044249	2.51E-22	30.05%
cellular macromolecule biosynthetic process	GO:0034645	2.84E-22	19.29%
cellular macromolecule metabolic process	GO:0044260	2.79E-05	34.28%
cellular metabolic process	GO:0044237	8.80E-05	53.06%
cellular nitrogen compound biosynthetic process	GO:0044271	1.64E-27	22.50%
cellular nitrogen compound metabolic process	GO:0034641	5.44E-13	34.14%
cellular protein metabolic process	GO:0044267	5.06E-14	14.29%
cellular respiration	GO:0045333	0.000833	0.98%
CTP biosynthetic process	GO:0006241	0.002933	0.14%
CTP metabolic process	GO:0046036	0.002933	0.14%
cytochrome complex	GO:0070069	0.000819	0.10%
cytoplasm	GO:0005737	1.08E-33	26.02%
cytoplasmic part	GO:0044444	3.68E-38	12.66%
drug metabolic process	GO:0017144	0.009584	0.06%
electron transport chain	GO:0022900	6.61E-06	0.56%
endopeptidase activity	GO:0004175	6.64E-11	1.92%
endoplasmic reticulum membrane	GO:0005789	0.007192	0.76%

endoplasmic reticulum part	GO:0044432	0.007824	0.82%
endoplasmic reticulum subcompartment	GO:0098827	0.007192	0.01%
energy coupled proton transport, down electrochemical gradient	GO:0015985	2.82E-05	0.41%
energy derivation by oxidation of organic compounds	GO:0015980	0.002933	1.18%
envelope	GO:0031975	3.62E-06	2.32%
establishment of protein localization to membrane	GO:0090150	0.006308	0.36%
gene expression	GO:0010467	1.34E-24	19.67%
generation of precursor metabolites and energy	GO:0006091	0.00312	1.94%
glycosyl compound biosynthetic process	GO:1901659	0.003474	1.47%
GTP biosynthetic process	GO:0006183	0.002933	0.05%
GTP metabolic process	GO:0046039	0.004078	0.10%
guanosine-containing compound biosynthetic process	GO:1901070	0.003474	0.13%
guanosine-containing compound metabolic process	GO:1901068	0.00475	0.29%
inner mitochondrial membrane protein complex	GO:0098800	4.77E-09	1.02%
inorganic cation transmembrane transport	GO:0098662	0.005418	0.93%
inorganic ion transmembrane transport	GO:0098660	0.005418	0.24%
intracellular	GO:0005622	6.95E-11	1.86%
intracellular non-membrane-bounded organelle	GO:0043232	1.52E-25	2.32%
intracellular organelle	GO:0043229	1.54E-13	41.18%
intracellular part	GO:0044424	6.64E-12	7.95%
intracellular ribonucleoprotein complex	GO:0030529	1.17E-36	19.92%
large ribosomal subunit	GO:0015934	0.011735	35.65%
macromolecule biosynthetic process	GO:0009059	4.04E-22	5.29%
macromolecule metabolic process	GO:0043170	2.06E-09	0.56%
membrane protein complex	GO:0098796	7.33E-05	14.01%
metabolic process	GO:0008152	2.66E-06	19.55%
mitochondrial ATP synthesis coupled electron transport	GO:0042775	8.46E-05	39.49%
mitochondrial electron transport, ubiquinol to cytochrome c	GO:0006122	0.002292	2.47%
mitochondrial envelope	GO:0005740	1.41E-06	75.39%
mitochondrial inner membrane	GO:0005743	5.56E-11	0.07%
mitochondrial membrane	GO:0031966	6.06E-07	0.04%
mitochondrial membrane part	GO:0044455	1.65E-08	0.90%

mitochondrial part	GO:0044429	7.73E-06	0.62%
mitochondrial protein complex	GO:0098798	3.26E-07	0.82%
mitochondrial proton-transporting ATP synthase complex	GO:0005753	0.000541	0.38%
mitochondrial proton-transporting ATP synthase complex, coupling factor F(o)	GO:0000276	0.000171	1.20%
mitochondrial respiratory chain	GO:0005746	2.09E-05	0.29%
mitochondrial respiratory chain complex I	GO:0005747	0.011735	0.07%
mitochondrial respiratory chain complex III	GO:0005750	0.003691	0.05%
mitochondrion	GO:0005739	1.05E-07	0.10%
monovalent inorganic cation transmembrane transporter activity	GO:0015077	0.001326	0.04%
monovalent inorganic cation transport	GO:0015672	0.002583	0.03%
NADH dehydrogenase (quinone) activity	GO:0050136	3.35E-07	2.16%
NADH dehydrogenase (ubiquinone) activity	GO:0008137	3.35E-07	1.39%
NADH dehydrogenase activity	GO:0003954	7.43E-08	1.82%
NADH dehydrogenase complex	GO:0030964	0.011735	0.36%
nitrogen compound metabolic process	GO:0006807	5.55E-11	0.30%
non-membrane-bounded organelle	GO:0043228	1.52E-25	0.37%
nuclear outer membrane-endoplasmic reticulum membrane network	GO:0042175	0.009968	0.04%
nucleobase-containing compound kinase activity	GO:0019205	0.003461	38.74%
nucleobase-containing small molecule metabolic process	GO:0055086	0.006061	8.41%
nucleoside biosynthetic process	GO:0009163	0.003474	0.77%
nucleoside diphosphate kinase activity	GO:0004550	0.001376	0.35%
nucleoside diphosphate metabolic process	GO:0009132	0.010552	4.92%
nucleoside diphosphate phosphorylation	GO:0006165	0.010552	1.44%
nucleoside monophosphate biosynthetic process	GO:0009124	5.13E-05	0.05%
nucleoside monophosphate metabolic process	GO:0009123	7.57E-06	0.70%
nucleoside phosphate biosynthetic process	GO:1901293	0.004965	0.60%
nucleoside phosphate metabolic process	GO:0006753	0.002072	1.49%

nucleoside triphosphate biosynthetic process	GO:0009142	2.66E-07	2.37%
nucleoside triphosphate metabolic process	GO:0009141	6.64E-08	2.60%
nucleotide biosynthetic process	GO:0009165	0.004965	4.21%
nucleotide metabolic process	GO:0009117	0.002072	0.65%
nucleotide phosphorylation	GO:0046939	0.010552	1.61%
odorant binding	GO:0005549	0.003461	2.56%
organelle	GO:0043226	2.00E-13	4.17%
organelle envelope	GO:0031967	3.62E-06	0.79%
organelle inner membrane	GO:0019866	7.70E-11	0.06%
organelle membrane	GO:0031090	0.00033	20.79%
organic substance biosynthetic process	GO:1901576	1.07E-21	1.26%
organic substance metabolic process	GO:0071704	3.17E-10	0.66%
organonitrogen compound biosynthetic process	GO:1901566	5.81E-38	2.00%
organonitrogen compound metabolic process	GO:1901564	1.61E-22	30.37%
oxidative phosphorylation	GO:0006119	0.000207	58.36%
oxidoreductase activity, acting on NAD(P)H	GO:0016651	1.82E-07	14.06%
oxidoreductase activity, acting on NAD(P)H, quinone or similar compound as acceptor	GO:0016655	3.35E-07	17.89%
oxidoreductase complex	GO:1990204	9.70E-05	0.25%
peptidase activity	GO:0008233	1.96E-06	0.80%
peptidase activity, acting on L-amino acid peptides	GO:0070011	1.63E-05	0.45%
peptidase complex	GO:1905368	0.008886	0.45%
peptide biosynthetic process	GO:0043043	8.74E-43	4.05%
peptide metabolic process	GO:0006518	3.60E-44	3.58%
primary metabolic process	GO:0044238	5.25E-11	0.45%
protein localization to membrane	GO:0072657	0.006308	5.77%
protein metabolic process	GO:0019538	7.85E-20	5.96%
protein-containing complex	GO:0032991	9.22E-29	53.74%
proteolysis	GO:0006508	8.37E-05	0.39%
proton transmembrane transport	GO:1902600	0.000585	18.49%
proton transmembrane transporter activity	GO:0015078	7.03E-05	5.22%
proton-transporting ATP synthase complex	GO:0045259	0.00193	0.49%
proton-transporting ATP synthase complex, coupling factor F(o)	GO:0045263	0.000171	0.22%

proton-transporting two-sector ATPase complex, proton-transporting domain	GO:0033177	0.004789	0.31%
proton-transporting V-type ATPase complex assembly	GO:0070070	0.004499	0.01%
purine nucleoside biosynthetic process	GO:0042451	0.003474	0.92%
purine nucleoside metabolic process	GO:0042278	0.00475	2.01%
purine nucleoside monophosphate biosynthetic process	GO:0009127	5.13E-05	1.04%
purine nucleoside monophosphate metabolic process	GO:0009126	7.57E-06	1.92%
purine nucleoside triphosphate biosynthetic process	GO:0009145	2.66E-07	0.49%
purine nucleoside triphosphate metabolic process	GO:0009144	6.64E-08	1.40%
purine nucleotide biosynthetic process	GO:0006164	2.01E-05	1.41%
purine nucleotide metabolic process	GO:0006163	2.61E-06	2.44%
purine ribonucleoside biosynthetic process	GO:0046129	0.003474	0.92%
purine ribonucleoside metabolic process	GO:0046128	0.00475	2.00%
purine ribonucleoside monophosphate biosynthetic process	GO:0009168	5.13E-05	1.04%
purine ribonucleoside monophosphate metabolic process	GO:0009167	7.57E-06	1.92%
purine ribonucleoside triphosphate biosynthetic process	GO:0009206	2.66E-07	0.49%
purine ribonucleoside triphosphate metabolic process	GO:0009205	6.64E-08	1.37%
purine ribonucleotide biosynthetic process	GO:0009152	8.61E-06	1.34%
purine ribonucleotide metabolic process	GO:0009150	1.27E-06	2.35%
purine-containing compound biosynthetic process	GO:0072522	2.26E-05	1.50%
purine-containing compound metabolic process	GO:0072521	2.88E-06	2.67%
pyrimidine nucleoside biosynthetic process	GO:0046134	0.002933	0.40%
pyrimidine nucleoside metabolic process	GO:0006213	0.002933	0.46%
pyrimidine nucleoside triphosphate biosynthetic process	GO:0009148	0.002933	0.22%
pyrimidine nucleoside triphosphate metabolic process	GO:0009147	0.002933	0.25%

pyrimidine nucleotide biosynthetic process	GO:0006221	0.005492	0.59%
pyrimidine nucleotide metabolic process	GO:0006220	0.005492	0.67%
pyrimidine ribonucleoside biosynthetic process	GO:0046132	0.002933	0.40%
pyrimidine ribonucleoside metabolic process	GO:0046131	0.002933	0.43%
pyrimidine ribonucleoside triphosphate biosynthetic process	GO:0009209	0.002933	0.14%
pyrimidine ribonucleoside triphosphate metabolic process	GO:0009208	0.002933	0.15%
pyrimidine ribonucleotide biosynthetic process	GO:0009220	0.002933	0.42%
pyrimidine ribonucleotide metabolic process	GO:0009218	0.002933	0.42%
respiratory chain	GO:0070469	2.09E-05	0.30%
respiratory chain complex	GO:0098803	2.09E-05	0.16%
respiratory chain complex I	GO:0045271	0.011735	0.04%
respiratory chain complex III	GO:0045275	0.003691	0.04%
respiratory electron transport chain	GO:0022904	1.95E-05	0.37%
ribonucleoprotein complex	GO:1990904	1.17E-36	5.29%
ribonucleoside biosynthetic process	GO:0042455	0.003474	1.42%
ribonucleoside metabolic process	GO:0009119	0.00475	2.52%
ribonucleoside monophosphate biosynthetic process	GO:0009156	5.13E-05	1.39%
ribonucleoside monophosphate metabolic process	GO:0009161	7.57E-06	2.27%
ribonucleoside triphosphate biosynthetic process	GO:0009201	2.66E-07	0.58%
ribonucleoside triphosphate metabolic process	GO:0009199	6.64E-08	1.46%
ribonucleotide biosynthetic process	GO:0009260	8.61E-06	1.73%
ribonucleotide metabolic process	GO:0009259	1.27E-06	2.75%
ribose phosphate biosynthetic process	GO:0046390	8.61E-06	1.78%
ribose phosphate metabolic process	GO:0019693	2.14E-06	3.03%
ribosome	GO:0005840	1.01E-40	4.20%
RNA binding	GO:0003723	0.003299	5.28%
rRNA binding	GO:0019843	0.000945	1.41%
serine hydrolase activity	GO:0017171	1.89E-11	1.24%
serine-type endopeptidase activity	GO:0004252	2.08E-12	0.81%
serine-type endopeptidase inhibitor activity	GO:0004867	0.003496	0.07%
serine-type peptidase activity	GO:0008236	1.89E-11	1.24%
signal peptidase complex	GO:0005787	0.005921	0.03%
signal peptide processing	GO:0006465	0.003016	0.05%

structural constituent of ribosome	GO:0003735	5.13E-51	2.68%
structural molecule activity	GO:0005198	3.58E-42	3.27%
sulfotransferase activity	GO:0008146	0.001722	0.09%
transferase activity, transferring sulfur-containing groups	GO:0016782	0.00222	0.43%
translation	GO:0006412	4.36E-43	5.69%
translation factor activity, RNA binding	GO:0008135	0.001206	0.91%
translational initiation	GO:0006413	0.001782	0.52%
UTP biosynthetic process	GO:0006228	0.002933	0.05%
UTP metabolic process	GO:0046051	0.002933	0.05%
vacuolar proton-transporting V-type ATPase complex assembly	GO:0070072	0.004499	0.01%

Table B. List containing all GO terms enriched in the DEGs set of *D. saccharalis* transcriptome.
